# Effects of Dry Herbal Supplementation on Microbiological Safety, Physicochemical Characteristics, Sensory Properties, and Shelf Life of Traditional Serbian Rolled *Pasta Filata* Cheese from Raw Cow’s Milk

**DOI:** 10.3390/microorganisms14030619

**Published:** 2026-03-10

**Authors:** Suzana Vidaković Knežević, Dragana Ljubojević Pelić, Nenad Popov, Slobodan Knežević, Jelena Vranešević, Miloš Pelić, Milica Živkov Baloš

**Affiliations:** 1 Scientific Veterinary Institute “Novi Sad”, 21000 Novi Sad, Serbia; dragana@niv.ns.ac.rs (D.L.P.); nenad.p@niv.ns.ac.rs (N.P.); slobodan.knezevic@niv.ns.ac.rs (S.K.); milos.pelic@fins.uns.ac.rs (M.P.); milica@niv.ns.ac.rs (M.Ž.B.); 2 Institute of Food Technology, University of Novi Sad, 21000 Novi Sad, Serbia

**Keywords:** Serbian *pasta filata* cheese, raw cow’s milk, artisanal cheese quality, herbs, shelf life

## Abstract

Rolled cheeses are a traditional specialty of the Vojvodina region in Serbia, produced through an artisanal process passed down across generations. This study evaluated the impact of the addition of selected herbs (a mixture of oregano and basil and chives added separately) on the microbiological, physicochemical, and sensory characteristics of rolled *pasta filata* cheese. Cheeses, both with and without herbs, were vacuum packed and stored at 4 °C for 60 days. The addition of oregano and basil significantly reduced aerobic mesophilic bacteria, *Enterobacteriaceae*, and *Escherichia coli*, while *Salmonella* spp. and *Listeria monocytogenes* remained undetectable throughout storage. Physicochemical analyses classified the cheeses as full-fat, semi-hard, with at least 45% milk fat in dry matter, and moisture in fat-free matter between 54% and 69%. All variants exhibited uniform shape, intact appearance, and a compact layered structure, while herbal-enriched cheeses developed a distinctive aroma and flavor. Sensory evaluation showed that all cheese types remained acceptable for up to 40 days, with minor deviations at day 60. Overall, the herbal addition enhanced sensory appeal, created new flavor profiles, and improved microbiological stability, demonstrating its potential as a natural strategy to extend the shelf life of traditional Serbian rolled *pasta filata* cheese.

## 1. Introduction

Stretched-curd cheeses, also known as *pasta filata* cheeses, are named after the distinctive production process involving curd stretching, which imparts their characteristic quality attributes [1]. Their origin is generally associated with the northern Mediterranean region, particularly Italy, the Balkan countries, Greece, Turkey, and Eastern Europe, where, despite modernization and industrialization, traditional methods of production are still preserved [2]. The most famous *pasta filata* cheeses are Mozzarella, Provolone, Scamorza, Caciocavallo, kashkaval and cheeses intended for pizza [3].

The production of rolled cheese in Vojvodina (an autonomous province in Northern Serbia), also known as “leaf cheese”, is believed to have originated in the late 1970s [4], although some oral traditions suggest that its history extends back more than a century. The recipe for rolled cheese has historically been preserved as part of a dowry and transmitted across generations, which accounts for the limited availability of written sources. The traditional production technology of rolled cheese is both unique and distinctive, relying on specific artisanal skills that are passed down within households from one generation to another. Traditionally, rolled cheeses are produced from raw cow’s milk, sheep’s milk, or a mixture of both [4].

The production of stretched-curd cheeses begins with the spontaneous fermentation of raw milk or the use of starter cultures. While rolled cheese can also be produced from pasteurized milk using thermophilic starter cultures, in the present study raw milk was used with spontaneous fermentation at 20 °C. When starter cultures are added, thermophilic lactic acid bacteria (LAB) are primarily used, such as *Streptococcus thermophilus* (formerly known as *Streptococcus salivarius* subsp. *thermophilus*) alone or in combination with *Lactobacillus delbrueckii* subsp. *bulgaricus* or *Lactobacillus helveticus* [5]. The resulting curd is cut and drained, followed by a unique process of thermal and mechanical treatment in hot water, whey, or brine, resulting in a cheese mass with an elastic-plastic consistency. The stretching of the cheese mass has a profound and multifaceted impact on the characteristics of the final cheese, affecting its structure as well as its microbiological, physicochemical, and sensory properties [2,3,5].

Rolled cheeses contain complex communities of microorganisms. The microbes come from raw milk, starter cultures, and other sources such as production equipment and surfaces in the facility. While microorganisms are essential for developing the flavor, texture, and overall quality of cheese by interacting with its constituents [6], milk and dairy products may harbor diverse microorganisms, including potential foodborne pathogens [7].

The incorporation of herbs and spices into cheese is an emerging approach in the dairy industry aimed at enhancing flavor, extending shelf life, and providing potential health benefits by reducing or eliminating pathogenic and spoilage bacteria, as well as improving nutritional and functional properties [8,9]. Traditional cheeses often incorporate herbs, as well as complex spice mixtures, to develop characteristic flavors and aromas. In these products, herbs are typically added to curd and thoroughly mixed without draining the whey to ensure uniform distribution [8], while certain varieties undergo additional surface treatment involving rubbing with selected herbs, spices, or their essential oils to further enhance their sensory properties [9].

Oregano, basil, and chives are flavoring herbs with notable functional properties. Oregano is rich in phenolic compounds responsible for its strong antioxidant and antimicrobial capacity. Basil also demonstrates broad-spectrum antimicrobial activity, and supports immune function, while exhibiting hypoglycemic, anti-inflammatory, and anxiolytic effects. Chives contribute digestive and anti-anemic benefits, enhance immune responses, and provide additional antioxidant activity [10].

Owing to these bioactive attributes, their incorporation into cheese may improve sensory quality and offer potential health-promoting effects. Building on this concept, the present study explores a modified technological approach in the traditional production of vacuum-packed rolled cheese by supplementing the cheese dough with selected dried herbs, including a mixture of oregano and basil, and chives. Although *pasta filata* cheeses with added herbs are widely produced, data on the application of such modifications to traditional rolled cheese from the Vojvodina region, particularly under vacuum packaging and refrigerated storage, remain limited.

Traditionally, rolled cheese is consumed fresh and is not subjected to standardized packaging, nor does it undergo a ripening process. It is typically marketed or consumed shortly after production, without storage in brine, resulting in a short shelf life of only a few days under refrigeration. Due to its high moisture content and rich microbial environment, rolled cheese is particularly susceptible to microbiological spoilage. In this context, vacuum packaging represents a technologically relevant approach to extend the storage period of fresh rolled cheese while maintaining its quality.

The primary objective of this study was to assess the potential of these selected dried herbs as natural fortifying agents in vacuum-packed rolled cheese. Specifically, the study evaluated their effects on the microbiological status, physicochemical properties, sensory attributes, and shelf life of rolled cheese during refrigerated storage at 4 °C. Overall, the research seeks to support the development of innovative, functionally enhanced versions of traditional rolled cheese.

## 2. Materials and Methods

### 2.1. Cheese Production

Raw milk used for cheese production was obtained during the morning milking from a single local dairy farm supplying the small-scale dairy plant in the Bačka region of Serbia. The farm maintained a Holstein Friesian herd with approximately 20 cows and an average daily milk production of 20–25 L per cow. Milk was transported to the processing facility under refrigerated conditions and used for cheese production on the same day, without the addition of preservatives. The farm was selected based on convenience sampling due to its long-term collaboration with the dairy plant.

At the facilities of a small dairy plant, three variants of *pasta filata*-type rolled cheeses were produced using a traditional manufacturing process: plain rolled cheese as the control (RC), rolled cheese with a mixture of equal parts of dried oregano and basil (RCOB), and rolled cheese with dried chives (RCC).

Fresh raw milk (Table 1) was allowed to spontaneously ferment at 20 °C for 24 h without any additives. The resulting coagulum was cut into grains approximately 10–20 mm in diameter and drained through a cheesecloth. The curd was then heated in a water bath at 70–80 °C with constant stirring and stretching using a wooden spoon until an elastic cheese dough consistency was achieved. The hot cheese dough was transferred to the work surface, followed by manual kneading, shaping, and stretching until a thickness of approximately 5 mm was obtained. The stretched cheese dough was hand-salted using sea salt. By repeatedly folding the dough, first one side inward, then the opposite side, a final width of approximately 30–40 cm was achieved. At this stage, to produce RCOB and RCC, the dried spicy herbs (a blend of oregano and basil, or chives) were added manually. The cheese dough was then rolled from both sides to form two rolls of approximately equal size. The rolls were wrapped in cotton cloth and stored at 4 °C for 24 h. Finally, the rolled cheeses were cut into 250 g portions, vacuum packed, and stored at 4 °C for up to 60 days.

### 2.2. Microbiological Analysis

Fresh raw cow’s milk was analyzed to determine the total bacterial count (TBC) (Table 1), in accordance with the national regulations governing raw milk quality. No additional microbiological analyses of raw milk, including the detection of specific foodborne pathogens, were performed, as they are not required by current legislation [11] and the focus of the study was not on raw milk safety.

The spicy herbs (oregano, basil, and chives), fresh curd, cheese dough, fresh rolled cheeses, and rolled cheeses stored for 20, 40, and 60 days were analyzed to enumerate TBC, *Enterobacteriaceae*, β-glucuronidase *Escherichia coli*, coagulase-positive staphylococci (CPS), and to detect *Salmonella* spp. and *Listeria monocytogenes*.

The TBC was determined on Plate Count Agar (PCA, CM0325, Oxoid, Basingstoke, UK) according to SRPS EN ISO 4833-1 [12], with aerobic incubation at 30 °C for 72 h. *Enterobacteriaceae* were enumerated on Violet Red Bile Glucose Agar (VRBGA, CM1082, Oxoid, UK) according to SRPS EN ISO 21528-2 [13], with aerobic incubation at 37 °C for 24 h. β-glucuronidase *E. coli* were enumerated on Tryptone Bile Glucuronide Agar (TBX, CM0945, Oxoid, UK) according to SRPS ISO 16649-2 [14], with aerobic incubation at 44 °C for 24 h. CPS were determined on Baird Parker agar (Biokar Diagnostics, Beauvais, France) according to SRPS EN ISO 6888-1 [15], with aerobic incubation at 37 °C for 24–48 h. Detection of *Salmonella* spp. and *L. monocytogenes* was performed according to SRPS EN ISO 6579-1 [16] and SRPS EN ISO 11290-1 [17], respectively.

### 2.3. Physicochemical Analysis

Raw milk samples were analyzed for fat and protein content (Table 1), while the rolled cheese variants (RC, RCOB, and RCC) were evaluated for total solids, fat, fat in dry matter (FDM), protein, pH, ash, sodium, sodium chloride, and water activity.

Total solids were determined by oven drying at 102 °C according to the ISO methodology [18]. Fat content was measured using the acido-butyrometric method [19], and FDM was calculated based on the moisture and fat content. Total nitrogen was determined by the Dumas method using a Rapid N Exceed Analyzer (Elementar, Langenselbold, Germany), and protein content was calculated using a conversion factor of 6.38 [20]. The pH of cheese samples was measured at 20 ± 0.5 °C with an electronic pH meter (Consort C 830, Turnhout, Belgium) calibrated with standard buffer solutions. Ash content was determined by dry ashing in a muffle furnace at 550 °C following AOAC procedures [21]. Sodium content was quantified using Agilent ICP-MS 7700 (Agilent Technologies, Santa Clara, CA, USA) after acid digestion of cheese samples in the Ethos system, Microwave Labstation (Milestone s.r.l., Sorisole, Italy) [22], and sodium chloride was calculated as sodium content × 2.5 (g/100 g). Water activity (a_w_) of the rolled cheeses was measured using the LabSwift-aw device (Novasina AG, Lachen, Switzerland), periodically calibrated according to the manufacturer’s instructions.

### 2.4. Sensory Analysis

Sensory evaluation of rolled cheeses (RC, RCOB, and RCC) was conducted in accordance with SOP 3-04-210 [23]. Assessments were performed on days 0, 20, 40, and 60 of storage by five trained panelists, all staff members of the Scientific Veterinary Institute “Novi Sad”, Serbia. Prior to evaluation, panelists participated in a session to harmonize the evaluation criteria and familiarize themselves with the assessed sensory attributes. Each panelist independently evaluated the samples in individual booths using a structured sensory quality assessment based on a five-point hedonic scale (1 = unacceptable, 5 = highly acceptable). The evaluated attributes included appearance (shape and surface integrity), color, texture (firmness and elasticity), aroma, and taste, which together contributed to the overall sensory acceptability of the cheeses.

Cheese samples were sliced to a diameter of 5–7 cm and a thickness of approximately 5 mm, and 20 g of each sample was presented on white plastic plates coded with three-digit random numbers. Samples were served at room temperature, and mineral water was provided for palate cleansing between assessments.

### 2.5. Statistical Analysis

Data presentation and analysis were performed using Microsoft Excel 2010 with the Data Analysis ToolPak, and the statistical software R version 3.2.2 (R Foundation for Statistical Computing, Vienna, Austria). All microbiological and physicochemical analyses were performed in triplicate, and sensory evaluation was conducted by five trained panelists. Reported values represent the mean of the replicates. Results were presented in tables and were subjected to descriptive statistical analysis, including calculation of means and standard deviations. Differences between treatments were assessed using the *t*-test, one-way ANOVA (Analysis of Variance), and Duncan’s multiple range test, with a significance level set at *p* < 0.05.

## 3. Results and Discussion

### 3.1. Microbiological Status of Spicy Herbs, Fresh Curd, Cheese Dough, and Rolled Cheeses

#### 3.1.1. Microbiological Status of Spicy Herbs

The microbiological status of the examined spicy herbs, including oregano, basil, and chives, is presented in Table 2. Among the spicy herbs tested, basil exhibited the lowest microbiological quality, showing a higher TBC compared to oregano and chives. Counts of *Enterobacteriaceae*, *E. coli*, and CPS were below quantification level (<1.00 log_10_ CFU/g), while *Salmonella* spp. and *L. monocytogenes* were not detected in any of the spicy herbs.

The TBC, *Enterobacteriaceae*, and *E. coli* counts reflect the hygienic quality of the spicy herb production. In the dataset, 35% of herb samples had TBC ≤ 3.0 log_10_ CFU/g, 36% ranged between 3.0 and 4.0 log_10_ CFU/g, and 27% exceeded 5.0 log_10_ CFU/g. Among pathogenic bacteria associated with herbs, *Salmonella* spp. is most prevalent (77% of cases), followed by *Bacillus cereus* (19.7%) [24]. Microbial contamination in herbs can occur at various stages, including field cultivation, drying, and storage. Nevertheless, herbs contain essential oils with antimicrobial properties that can inhibit microbial growth [25].

#### 3.1.2. Microbiological Status of Fresh Curd and Cheese Dough

The microbiological status of fresh curd and cheese dough is presented in Table 3. Although the raw milk used in this study exhibited relatively low total bacterial counts, spontaneous fermentation at 20 °C for 24 h was successfully initiated. This can be explained by the presence of indigenous LAB and other microorganisms in raw milk, which, even at low initial numbers, can rapidly proliferate under favorable temperature and nutrient conditions [26]. The slow but continuous growth of these autochthonous microorganisms during 24 h incubation allowed them to dominate the curd microbiota, resulting in very high total bacterial counts in fresh curd and cheese dough (Table 3). Other microbial groups such as *Enterobacteriaceae*, *E. coli*, and CPS may also be found in raw milk, due to their presence on teat surfaces and milking equipment, and grow in the fermentation phase [26]. Steaming the curd in a water bath at 70–80 °C to obtain the cheese dough resulted in a significant (*p* < 0.05) reduction in TBC by 1.38 log_10_ CFU/g and *Enterobacteriaceae* by 1.00 log_10_ CFU/g. The steaming process did not significantly reduce *E. coli* or CPS. Foodborne pathogens, *Salmonella* spp. and *L. monocytogenes*, were not detected in any fresh curd or cheese dough samples.

A greater reduction in TBC (2.2 ± 1.1 log_10_ CFU/g), yeasts and molds (2.6 ± 0.9 log_10_ CFU/g), and *E. coli* (4.0 ± 1.0 log_10_ CFU/g) was reported by Lehotová et al. [27] during the production of *pasta filata* cheeses made from raw cow’s and sheep’s milk in the vicinity of Bratislava, Slovak Republic.

The curd used for Fior di Latte di Agerola, a traditional cheese from Southern Italy (Naples region), exhibited slightly higher counts of *Enterobacteriaceae* (6.61 ± 0.16 log_10_ CFU/g) and CPS (5.0 ± 0.0 log_10_ CFU/g) [28] compared to the fresh curd used in the production of rolled cheeses. Fior di Latte di Agerola is produced from raw cow’s milk by combining freshly collected morning milk with cooled evening milk, followed by spontaneous fermentation, cutting, draining, maturation at room temperature for 8 to 10 h, a final stretching in water at 80 °C to 90 °C, and shaping into its final form [29].

During the production of the traditional Italian *pasta filata* cheese Casizolu, made from raw cow’s milk of the Sardo-Modicana and/or Bruno-Sarda breeds with the addition of natural whey starter, the number of TBC in the cheese dough after steaming at 70 °C to 80 °C for 5 min was found to be 6.0 ± 0.6 log_10_ CFU/g and 7.9 ± 0.7 log_10_ CFU/g [29], which is comparable to the results obtained during the production of rolled cheeses. The number of *Enterobacteriaceae* in Casizolu stretched curd was slightly lower (1.8 ± 1.1 log_10_ CFU/g) compared to that found during the production of rolled cheeses. The same trend was observed for *E. coli* (<3 log_10_ CFU/g), and CPS (<1 log_10_ CFU/g) [29].

#### 3.1.3. Microbiological Status of Rolled Cheeses

The microbiological status of the rolled cheese variants (RC, RCOB, and RCC) during storage is presented in Table 4. At the beginning of storage, the TBC in RCOB was significantly (*p* < 0.05) lower than in RC (by 0.21 log_10_ CFU/g) and RCC (by 0.26 log_10_ CFU/g). This trend persisted throughout the 60-day storage period, with TBC in RCOB remaining 0.35 log_10_ CFU/g lower than RC and 0.37 log_10_ CFU/g lower than RCC at day 60.

Similarly, the initial *Enterobacteriaceae* count in RCOB was significantly (*p* < 0.05) lower compared to RC and RCC. By day 20, a substantial decrease in *Enterobacteriaceae* was observed in RCOB (by 2.37 log_10_ CFU/g compared to RC and by 1.87 log_10_ CFU/g compared to RCC), and this pattern was maintained until the end of storage, when *Enterobacteriaceae* counts in RCOB were 0.98 log_10_ CFU/g and 1.09 log_10_ CFU/g lower than in RC and RCC, respectively.

The number of *E. coli* in RCOB was significantly (*p* < 0.05) reduced from day 20 of storage and remained below the quantification level (<1.00 log_10_ CFU/g) until the end of the storage period.

CPS counts did not differ significantly among the cheese variants (*p* > 0.05) throughout the storage period.

Foodborne pathogens, *Salmonella* spp. and *L. monocytogenes*, were not detected in any of the rolled cheese variants during the 60-day storage period.

The high initial levels of TBC, *Enterobacteriaceae*, *E. coli*, and CPS observed in rolled cheeses at day 0, despite the relatively low microbial load in raw milk, are likely due to a combination of factors. During cheese production, including spontaneous fermentation, manual stretching, shaping, and rolling, microbial contamination can occur from the processing environment, equipment, and handling practices, which may temporarily increase the microbial load in the final product.

Overall, microbiological analysis of rolled cheeses showed that the addition of a herb mixture of oregano and basil resulted in a significant reduction of certain microorganisms, including TBC, *Enterobacteriaceae*, and *E. coli*. This reduction is attributed to the essential oils present in herbs. The antimicrobial activity of essential oils has been demonstrated in numerous studies. Previous research has shown that essential oils of oregano and basil possess strong antimicrobial effects, both in vitro and in food models, including various types of cheeses [30,31,32,33,34,35,36,37]. However, it should be considered that the antimicrobial activity of a given herb has limitations in food models, due to interactions with food compounds and the influence of external and internal factors [38,39]. Antimicrobial components may lose activity in the presence of fats and proteins in cheeses, which can coat microorganisms and protect them from antimicrobial agents [38], as well as due to sensitivity to temperature, pH, or light [9].

Herbs are primarily added to cheeses to improve or enrich flavor, but their antimicrobial and antioxidant properties also have a beneficial effect on human health [39,40].

Although no foodborne pathogens were detected, the high initial microbial loads highlight the importance of food safety measures in artisanal *pasta filata* cheese production. Monitoring raw milk quality, proper sanitation of equipment, careful handling during curd processing and routine microbiological testing of final products are recommended to reduce potential risks. The inclusion of antimicrobial herbs, such as oregano and basil can further inhibit microbial proliferation, contributing to the overall safety of the cheeses.

### 3.2. Physicochemical Characteristics of Rolled Cheeses

The results of the physicochemical parameters of RC, RCOB, and RCC are presented in Table 5. The total solids content ranged from 50.21% to 53.61%, with a statistically significant difference (*p* < 0.05) between the rolled cheeses observed only on day 0, when the rolled cheeses with herbs (RCOB and RCC) had slightly lower dry matter values compared to the RC, that is, not less than 20%, which is in accordance with the requirements prescribed by Serbian regulations [41]. No significant differences (*p* > 0.05) were observed among cheeses containing different types of dried spices suggesting that the type of spice did not differentially influence the initial total solids content. During storage (days 20, 40, and 60), no statistically significant differences in total solids content were detected among the different cheese types. The addition of oregano, basil, or chives may enhance water-binding capacity and consequently contribute to reduced variation in total solids [40,42]. This is in agreement with our results and the statistically significant difference (*p* < 0.05) observed on day 0, where the rolled cheese without spices (RC) exhibited a higher percentage of total solids compared to cheeses supplemented with oregano and basil, as well as cheese containing dried chives. A slightly higher total solids content (56.02%) was observed in Pirot’s kashkaval, a type of *pasta filata* cheese made from cow’s milk originating from Stara Planina (southeastern part of Serbia) [43]. Regarding the effect of storage time, in the control group the total solids content changed significantly over time, with the lowest value recorded on day 60 of storage. However, no statistically significant difference was observed between days 20 and 60. In rolled cheese supplemented with a mixture of basil and oregano, a statistically significant decrease (*p* < 0.05) in total solids content was also observed, with the lowest value measured on day 60. Nevertheless, no statistically significant differences were found among days 20, 40, and 60, nor between days 0 and 20 of storage. In cheese supplemented with dried chives (RCC), no statistically significant variations in total solids content were recorded during storage. Changes in total solids during cheese storage are primarily governed by moisture dynamics within the cheese matrix [44]. During the first three weeks of refrigerated storage, water redistribution occurs within the cheese, reflected in reduced whey release and an apparent increase in water-holding capacity [45]. Similarly, in our study, no statistically significant difference in total solids content was observed in the control group between 20 and 60 days of storage, which may indicate that most compositional changes occurred during the early storage period. The mechanisms underlying this shift in water mobility, as well as the precise role of water in cheese functionality, remain unclear [46].

Vacuum packaging represents a closed system that prevents moisture evaporation [47,48]. Therefore, in vacuum-packaged cheese stored at 4 °C, no true loss of total solids is expected. Instead, a gradual redistribution of water may occur, leading to relative changes in the concentration of cheese constituents. During storage, water may migrate within the protein–fat matrix and form surface exudate [49], which can result in apparent variations in measured dry matter. If this exudate is not analyzed together with the cheese sample, the calculated total solids content may be overestimated. The addition of dried herbs may also influence chemical stability during storage by modifying water retention and microstructural organization, thereby contributing to minor differences among treatments. If herbal particles enhance water retention in the cheese matrix, the measured total solids may appear lower due to the higher moisture fraction, even though the absolute amount of solid components remain unchanged [40,50]. Masotti et al. [51] observed a random trend in moisture variation and only a slight decrease in moisture content after 60 days of storage of vacuum-packaged goat cheese. Water activity remained similar among treatments during the declared storage period and decreased only thereafter. Similarly, Frau et al. [52] reported that both vacuum packaging and storage time influenced moisture content, color variation, and rind formation in artisanal goat cheeses stored under refrigeration. Vacuum-packed cheeses exhibited improved visual appearance and higher water retention. However, vacuum packaging is not equally suitable for all cheese types [48,53]. While it can inhibit or reduce the growth of certain microorganisms, it may also produce undesirable effects on color, flavor, and texture, or promote the appearance of surface moisture due to a migration of water from the interior to the surface [53,54,55]. A significant difference in moisture loss has been observed between packaging systems, with vacuum packaging resulting in a markedly smaller decrease in moisture compared to non-vacuum storage [56]. By reducing moisture loss, vacuum packaging provides an economic advantage when cheese is sold by weight. On the other hand, Inácio et al. [53] reported that vacuum packaging had no effect on the moisture content of the cheese. Moisture content is also influenced by technological factors, particularly the stretching pH during *pasta filata* manufacture. Maldonado et al. [57] demonstrated that higher stretching pH values are associated with lower final moisture content in *pasta filata* cheese. The authors explained this relationship by noting that at higher stretching pH values the temperature is higher, which leads to greater whey loss during the stretching process and consequently reduces water retention in the cheese.

The fat content in rolled cheeses during storage ranged between 21.50% and 25.25%. A similar level of fat content has been reported in various *pasta filata* cheeses, including rolled cheese made from cow’s milk (24.50%), rolled cheese with the addition of 10% donkey’s milk (21.75%) [58], kashkaval cheeses (ranging from 20% to 29.50%) [59], Mozzarella di Bufala Campana (21%), low-moisture Mozzarella (24%), and Caciocavallo Pugliese (24%) [1]. Fat content in our study was significantly higher on day 0 in the control group (RC), while no significant difference was observed between rolled cheese supplemented with a mixture of basil and oregano (RCOB) and rolled cheese with added chives (RCC). This initial difference in fat content may be associated with variations in water retention, as the addition of dried spices could influence the water-binding capacity [40,42] and consequently affect fat concentration. On day 20, fat content was significantly higher in the RC and RCOB groups. The same pattern was observed on day 40; however, no statistically significant difference was detected between the RCOB and RCC groups at that time. On day 60 of storage, the fat percentage was again highest in the control group, although the difference was not statistically significant. During storage, no statistically significant changes in fat content were observed within the RC or RCOB groups. A significant difference was detected only in the RCC group, where a lower fat percentage was measured on day 40 compared to days 0 and 60 of storage. Regarding fat in dry matter, its percentage remained constant both among different cheese types and throughout storage, with no statistically significant differences observed between treatments or across storage periods. These results indicate that the observed changes in fat content are more likely associated with changes in moisture, structural redistribution within the cheese matrix, and sampling variability rather than a true loss of fat. Frau et al. [52] reported that fat content significantly decreased over storage time in vacuum-packaged artisanal goat cheese. In contrast, Todaro et al. [60] found that vacuum packaging of *pasta filata* cheese did not influence changes in fat content. Lipolysis in vacuum-packaged cheese proceeds slowly and involves the hydrolysis of triglycerides into free fatty acids and glycerol [61]. Fuentes et al. [62] did not observe notable lipolytic activity during storage of cheese under vacuum packaging. It is well known that herbs contain various bioactive compounds with antioxidant and antimicrobial properties [63], which may potentially slow lipolytic and proteolytic processes. This suggests that cheeses supplemented with herbs could exhibit a more stable chemical composition during storage compared to the control cheese. However, Kalit et al. [8] reported that both proteolysis and lipolysis increased with higher proportions of spices added to cheese. Although lipolysis alters lipid composition and contributes to flavor development, it does not lead to a significant reduction in total fat mass. Herbal addition may influence fat distribution by modifying matrix structure and water retention, as well as through bioactive compounds with mild antimicrobial activity that can affect lipolytic processes. Kurćubić et al. [64] did not observe changes in fat content in Pirot kashkaval following the addition of plant extracts.

The FDM in rolled cheeses ranged between 41.79% and 48.29%, with no statistically significant difference (*p* > 0.05) between rolled cheeses during the storage. Based on these results, the rolled cheeses can be classified as full-fat cheeses (≥45% and <60%) [41]. A slightly higher FDM was observed in Pirot’s kashkaval (54.89%) [43] and Telita cheese (ranging from 49.32% to 54.94%) originating from Venezuela [57].

The protein content in rolled cheeses during 60 days of storage varied between 20.52% and 25.35%. A statistically significant difference (*p* < 0.05) in protein content was observed only on day 60 of storage, when the protein level was slightly higher in RCC compared to RC. In the control group, protein content was significantly lower (*p* < 0.05) on days 20 and 60 of storage. The lowest protein content was measured on day 40; however, this value did not differ significantly from that recorded on day 20. In the control group, protein content decreased significantly (*p* < 0.05) on days 20 and 60 of storage. Although the lowest value was recorded on day 40, it did not differ significantly (*p* > 0.05) from that measured on day 20, indicating moderate changes rather than a continuous downward trend. These variations may be associated with moisture redistribution, proteolytic activity, or structural changes within the cheese matrix during storage. In the RCOB group, the lowest protein content was observed on day 20, with no significant differences (*p* > 0.05) compared to days 0 and 60. The highest value was measured on day 40; however, it did not differ significantly (*p* > 0.05) from the initial value, suggesting overall compositional stability throughout storage. In the RCC group no statistically significant changes (*p* > 0.05) in protein content were observed during storage under vacuum conditions at refrigeration temperature. An almost identical protein level was observed in rolled cheese made from cow’s milk (20.95%), rolled cheese with the addition of 10% donkey’s milk (21.28%), rolled cheese with the addition of 20% donkey’s milk (23.19%) [58], high-moisture (22%) and low-moisture (21%) Mozzarella, Caciocavallo Pugliese (23.5%), and Provolone Valpadana (24%) [1]. Conversely, a higher protein content was observed in Mexican *pasta filata* cheese Oaxaca (ranging from 26.5% to 31.6%) [65], Ragusano (30%), Caciocavallo Silano (33%) [1], and Telita cheese (ranging from 37.49% to 43.02%) [57], while a lower protein content was observed in Mozzarella di Bufala Campana (19%) [1], and Pirot’s kashkaval (19.30%) [43]. The increase in protein content may be associated with bioactive compounds present in herbs, particularly phenolic compounds, which may enhance protein stability [66,67]. During refrigerated vacuum storage, proteolysis proceeds slowly due to residual rennet activity and microbial enzymes, resulting in the breakdown of caseins into peptides and free amino acids [68]. This process does not represent a true loss of total protein from the cheese matrix, but rather a transformation into more soluble nitrogen fractions. Apparent decreases in measured protein content may therefore reflect redistribution of soluble components, minor losses of surface exudate, and changes in moisture content rather than an actual reduction in total protein mass. Changes in the microstructure of Mozzarella during storage indicate that the protein network is not immediately stabilized after stretching and molding but undergoes gradual structural reorganization [69]. The presence of dried herbs may further influence water binding and cheese microstructure and exert mild antimicrobial effects, potentially modifying the rate of proteolysis. Kalit et al. [8] reported that both proteolysis and lipolysis increase with higher proportions of spices added to cheese. Kurćubić et al. [64] did not report changes in protein content in Pirot kashkaval following the addition of plant extracts. Fuentes et al. [62] did not observe measurable proteolytic activity during vacuum storage. Todaro et al. [60] found that vacuum packaging of *pasta filata* cheese did not significantly affect protein content. Protein content is also influenced by technological factors, particularly stretching pH. Maldonado et al. [57] demonstrated that protein levels varied significantly at different stretching pH values in *pasta filata* cheese, with the highest protein contents observed in cheeses stretched at pH 5.2 and 5.5. The authors attributed this to a substantial reduction in fat content at these pH levels, resulting in a relative increase in protein concentration.

The pH ranged from 4.89 and 5.78 across the different rolled cheeses. A similar pH value was observed in Mozzarella di Bufala Campana, Ragusano, Provolone Valpadana (5.3), low-moisture Mozzarella, Caciocavallo Silano, Caciocavallo Pugliese (5.4), and in high-moisture Mozzarella (5.7) [1]. In contrast, a higher pH value was observed in Oaxaca (ranging from 6.2 to 6.6) [65], while a lower pH value was observed in Pirot’s kashkaval (3.52) [43]. The pH value on days 0, 20, and 60 of storage was highest in the RC group, followed by the RCOB group, and lowest in the RCC group, with statistically significant differences (*p* < 0.05) observed among treatments. On day 40 of storage, the highest pH value was recorded in the RCC group, followed by RC, while the lowest value was observed in the RCOB group (*p* < 0.05). Regarding storage time, in all three cheese types the pH value was highest on day 0, decreased to its lowest level on day 20, and then gradually increased on days 40 and 60 of storage. The observed pH fluctuations during storage can be interpreted in relation to microbial activity and ongoing biochemical changes in the cheese matrix. Residual lactic acid bacteria may continue limited fermentation during refrigerated storage, contributing to acid production and localized pH decreases [42], while proteolytic reactions generate peptides and amino compounds that may partially buffer acidity and promote slight pH increases over time [70]. Increased proteolytic activity is associated with a rise in pH and a decrease in water activity. Proteolysis contributes to textural changes in the cheese curd by disrupting the protein network, lowering water activity through the binding of water to newly exposed carboxyl and amino groups, and promoting an increase in pH [60]. The lower microbial counts observed in the herb-supplemented cheeses, particularly in RCOB, suggest that the antimicrobial activity of oregano and basil essential oils may have moderated microbial metabolism, contributing to differences in pH evolution among treatments. Kurćubić et al. [64] did not report changes in pH in Pirot kashkaval following the addition of plant extracts. In vacuum-packaged cheese, microbial acidification and proteolytic buffering occur slowly at the same time, which may explain the small but significant pH changes observed during storage [53]. Masotti et al. [51] did not observe statistically significant variations in pH during refrigerated storage of goat cheese under different storage conditions. Similarly, in the study conducted by Franco et al. [48] pH remained nearly constant at around 5.8 throughout storage, and vacuum packaging had no effect. The authors attributed this stability to lactose metabolism and lactic acid formation during the early stages of cheese manufacture, as well as to a moderate level of proteolysis. In contrast, Fuentes et al. [62] reported a significant decrease in pH during 24 days of refrigerated storage of vacuum-packaged Oaxaca cheese (a Mexican *pasta filata* cheese), with pH declining to approximately 5.0.

The ash content ranged from 2.37% to 3.39% among the rolled cheeses, with no statistically significant differences (*p* > 0.05) observed throughout the storage period. Similarly, an ash content of 3.62% was reported for Pirot’s kashkaval [43].

The sodium and sodium chloride contents ranged from 0.43 g/100 g to 0.83 g/100 g, and from 1.08 g/100 g to 2.06 g/100 g, respectively. No statistically significant differences (*p* > 0.05) were observed between the rolled cheeses throughout the storage period in terms of these parameters. The sodium chloride content in *pasta filata* cheeses was reported to range from 0.53% to 3.9% [1,43,57,58,59,65].

The a_w_ ranged between 0.945 and 0.969. Similarly, Palmito cheese, a stretched unripe cheese from Costa Rica, exhibited an a_w_ ranging from 0.967 to 0.988 [71]. In our study, water activity did not differ significantly (*p* < 0.05) among the different cheese types on days 0 and 40 of storage. On day 20, it was significantly higher in the RCC group compared to the RCOB and RC groups, between which not statistically significant (*p* > 0.05) difference was observed. On day 60, the highest water activity was recorded in the RCOB group, with a statistically significant difference (*p* < 0.05) compared to the RC group, but not (*p* > 0.05) compared to the RCC group. These variations in water activity may be associated with differences in moisture retention and redistribution within the cheese matrix during storage.

The results of this study, as well as those of previous research, indicate that the physicochemical characteristics of *pasta filata* cheeses vary and depend on the technological process of cheese production, the quality of the milk, the fermentation method, the stretching temperature and time, as well as the ripening period. For traditional rolled cheese, the absence of a ripening stage and the specific stretching and folding procedures, together with vacuum storage, play a key role in maintaining physicochemical parameters relatively stable during refrigerated storage, thereby supporting the technological suitability of this approach for preserving the intrinsic quality of rolled cheese.

### 3.3. Sensory Characteristics of Rolled Cheeses

**Figure 1:**
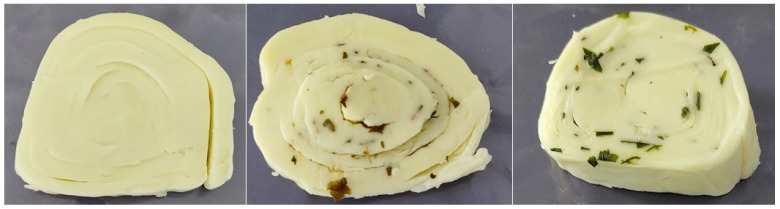
Cross-sections of rolled cheese variants: plain rolled cheese as the control (RC) (**left**), rolled cheese with a mixture of equal parts of dried oregano and basil (RCOB) (**middle**), and rolled cheese with dried chives (RCC) (**right**) on day 0 of evaluation.

The results of the sensory properties of RC, RCOB, and RCC are presented in Table 6. On day 0 of evaluation, all rolled cheese variants, including RC, RCOB, and RCC, were uniform in shape and size, free of deformation and damage, with a clearly defined layered structure visible in cross-section (Figure 1), receiving high appearance scores (mean values above 4.60 on a five-point hedonic scale). In the cross-sections of the rolled cheeses with added spicy herbs, a mixture of oregano and basil or chives was visible. The color of the rolled cheeses was white with a slight yellowish hue, while the herbs exhibit characteristic green to dark green shades. The texture of the rolled cheeses was semi-hard. They had a mild, milky-sour aroma. The basic taste of the rolled cheeses was pleasant and milky, not overly salty or sour, and without any bitterness. The RCOB had a distinctive oregano and basil flavor, whereas the RCC had a characteristic taste reminiscent of garlic. During chewing, a distinctive aroma was released and retained, accompanied by a creamy, melt-in-mouth texture.

**Figure 2:**
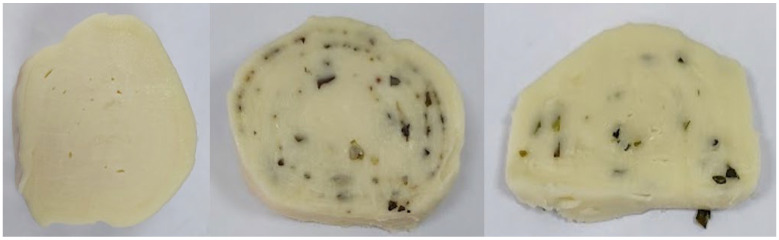
Cross-sections of the plain rolled cheese (RC) (**left**), the rolled cheese with a spice blend of oregano and basil (RCOB) (**middle**), and the rolled cheese with chives (RCC) (**right**) on day 60 of evaluation.

The characteristic sensory properties of the RC, RCOB, and RCC were preserved on both the 20th and 40th days of storage at 4 °C under vacuum. During the sensory evaluation of the RC, RCOB, and RCC on the 60th day of storage, slight deviations from the defined characteristics were observed (Figure 2). Slicing the rolled cheeses became more difficult due to the cheese sticking to the knife blade. In all three types of rolled cheeses, the slices were considerably irregular in shape, and the layered structure was mostly disrupted. The visibility of the added herbs in the cross-section was reduced. These changes were reflected in a decrease in appearance and texture scores; however, mean hedonic values remained above 3.00 on the five-point scale, indicating that the products were still considered acceptable. The color of the rolled cheeses remained unchanged, white with a yellowish hue, as did the color of the herbs, ranging from green to dark green. Additionally, the texture of the rolled cheeses was compromised. When pressed with finger, the cheeses exhibited a softer, dough-like, and slightly sticky consistency. Despite these changes, the rolled cheeses retained their characteristic milky-sour aroma. The basic flavor of the RC was slightly altered by the presence of mild bitterness. This bitterness was masked by the added herbs: in the RCOB, the distinctive oregano and basil flavor was dominant, while in the RCC, a characteristic garlic-like aroma prevailed. While chewing, a sticky, melt-in-the-mouth texture was perceived.

The use of herbs in cheese production is an ancient practice, most often associated with local tradition. Herbs, whether fresh or dried, are primarily used to impart specific flavor or aromas to cheese and to extend its shelf life [9,72,73].

As with rolled cheese containing a spice blend and those with added chives, masking of the basic aroma and flavor has also been reported in cheeses with added rosemary [74], parsley, dill, pepper, and garlic [25], with these products being well accepted by sensory panelists. The addition of the herbs such as nettle, parsley, mint, and arugula to Kashar cheese positively influenced the color and texture of this Turkish semi-hard cheese [73]. In contrast, no influence of the added herbs on color and texture was observed in the RCOB and RCC.

## 4. Conclusions

In conclusion, microbiological analysis of the rolled cheeses showed that the addition of a spicy herb mixture of oregano and basil resulted in a significant reduction of certain microorganisms, including TBC, *Enterobacteriaceae*, and *E. coli*. Furthermore, the addition of the oregano and basil herb mixture, as well as chives, influenced the sensory properties of the rolled cheeses by enhancing their aroma. In contrast, the addition of herbs did not affect the physicochemical properties of the rolled cheeses. The observed variations were instead a consequence of traditional artisanal production using raw milk that had not undergone prior processing. Based on the evaluation of microbiological, physicochemical, and sensory characteristics of rolled cheeses with and without the addition of herbs, vacuum-packed and stored for 60 days, it can be concluded that these cheeses maintain their characteristic properties for up to 40 days, which can be considered their shelf life. These findings highlight the potential of selected herbs as natural adjuncts for improving the microbiological quality and sensory appeal of traditionally produced rolled cheeses, particularly in small-scale and artisanal production systems.

Future research could focus on optimizing the types and concentrations of added spicy herbs. Additionally, studies could investigate the impact of different milk treatments, such as pasteurization and microfiltration, on the physicochemical and microbiological stability of these cheeses. Long-term storage studies under various packaging conditions could provide more precise information on shelf life, while consumer preference analyses could help determine market acceptance of flavored rolled cheeses. Finally, exploring the use of natural preservatives or starter cultures in combination with herbs may offer an effective strategy for improving both safety and quality in artisanal cheese production.

## Figures and Tables

**Table 1:** Characteristics of raw cow’s milk used in the production of rolled cheeses (means ± SD).

Parameter	Raw Cow’s Milk
Fat (%)	3.95 ± 0.18
Protein (%)	3.31 ± 0.25
Total bacteria count (log_10_ CFU/mL)	4.76 ± 4.39

**Table 2:** Microbiological status of oregano, basil, and chives.

Parameter	Oregano	Basil	Chives
TBC (log_10_ CFU/g)	3.52 ± 0.16	4.74 ± 0.39	3.76 ± 0.14
*Enterobacteriaceae* (log_10_ CFU/g)	<1.00	<1.00	<1.00
*E. coli* (log_10_ CFU/g)	<1.00	<1.00	<1.00
CPS (log_10_ CFU/g)	<1.00	<1.00	<1.00
*Salmonella* spp. (25 g)	Not detected	Not detected	Not detected
*L. monocytogenes* (25 g)	Not detected	Not detected	Not detected

The results are presented as means ± SD.

**Table 3:** Microbiological status of fresh curd and cheese dough.

Parameter	Fresh Curd	Cheese Dough
TBC (log_10_ CFU/g)	9.26 ± 0.03 ^a^	7.88 ± 0.02 ^b^
*Enterobacteriaceae* (log_10_ CFU/g)	5.17 ± 0.00 ^a^	4.17 ± 0.11 ^b^
*E. coli* (log_10_ CFU/g)	4.09 ± 0.05	3.77 ± 0.07
CPS (log_10_ CFU/g)	3.07 ± 0.07	2.45 ± 0.15
*Salmonella* spp. (25 g)	Not detected	Not detected
*L. monocytogenes* (25 g)	Not detected	Not detected

Results are expressed as means ± SD. Values within the same row marked with different lowercase superscript letters (^a,b^) indicate statistically significant differences (*p* < 0.05).

**Table 4:** Microbiological status of rolled cheese variants (plain rolled cheese as the control (RC), rolled cheese with a mixture of equal parts of dried oregano and basil (RCOB), and rolled cheese with dried chives (RCC)) at 0, 20, 40, and 60 days of storage.

Microorganism	Rolled Cheese	Storage Time (Days)			
		0	20	40	60
TBC (log_10_ CFU/g)	RC	7.95 ± 0.08 ^aA^	7.90 ± 0.12 ^aA^	7.59 ± 0.18 ^aB^	7.74 ± 0.22 ^aAB^
	RCOB	7.74 ± 0.08 ^bA^	7.49 ± 0.39 ^bAB^	7.14 ± 0.20 ^bB^	7.39 ± 0.12 ^bAB^
	RCC	8.00 ± 0.14 ^aAB^	8.29 ± 0.16 ^aA^	7.87 ± 0.33 ^aB^	7.76 ± 0.14 ^aB^
*Enterobacteriaceae* (log_10_ CFU/g)	RC	2.81 ± 0.13 ^aA^	2.95 ± 0.53 ^aA^	1.80 ± 0.15 ^aB^	1.68 ± 0.50 ^aB^
	RCOB	2.15 ± 0.04 ^bA^	0.58 ± 0.68 ^bB^	0.58 ± 0.68 ^bB^	0.70 ± 0.81 ^bB^
	RCC	2.62 ± 0.16 ^aA^	2.45 ± 0.25 ^aA^	1.73 ± 0.11 ^aB^	1.79 ± 0.18 ^aB^
*E. coli* (log_10_ CFU/g)	RC	3.08 ± 0.43 ^aA^	2.37 ± 0.12 ^aB^	1.57 ±0.30 ^aC^	1.30 ± 0.24 ^aC^
	RCOB	1.32 ± 0.44 ^cA^	<1.0 ^bB^	<1.0 ^bB^	<1.0 ^bB^
	RCC	2.21 ± 0.18 ^bA^	2.28 ± 0.06 ^aA^	1.52 ± 0.60 ^aAB^	1.15 ± 0.83 ^aB^
CPS (log_10_ CFU/g)	RC	1.27 ± 0.37 ^aB^	2.18 ± 0.10 ^aA^	2.40 ± 0.32 ^aA^	2.07 ± 0.78 ^aA^
	RCOB	0.58 ± 0.68 ^aB^	2.06 ± 0.27 ^aA^	1.90 ± 0.32 ^aA^	1.75 ± 0.70 ^aA^
	RCC	0.70 ± 0.86 ^aB^	2.27 ± 0.31 ^aA^	2.10 ± 0.48 ^aA^	1.95 ± 0.66 ^aA^
*Salmonella* spp. (25 g)	RC	Not detected	Not detected	Not detected	Not detected
	RCOB	Not detected	Not detected	Not detected	Not detected
	RCC	Not detected	Not detected	Not detected	Not detected
*L. monocytogenes* (25 g)	RC	Not detected	Not detected	Not detected	Not detected
	RCOB	Not detected	Not detected	Not detected	Not detected
	RCC	Not detected	Not detected	Not detected	Not detected

Results are presented as means ± SD. Values within the same column marked with different lowercase superscript letters (^a,b,c^) indicate statistically significant differences (*p* < 0.05) between different rolled cheeses. Values within the same row marked with different uppercase letters (^A,B,C^) indicate statistically significant differences (*p* < 0.05) among storage time for the same cheese.

**Table 5:** Physicochemical status of rolled cheese variants (plain rolled cheese as the control (RC), rolled cheese with a mixture of equal parts of dried oregano and basil (RCOB), and rolled cheese with dried chives (RCC)) at 0, 20, 40, and 60 days of storage.

Parameters	Rolled Cheese	Storage Time (Days)			
		0	20	40	60
Total solids content (%)	RC	53.61 ± 0.26 ^aA^	51.85 ± 0.60 ^aBC^	52.44 ± 0.28 ^aB^	51.26 ± 0.13 ^aC^
	RCOB	51.98 ± 0.28 ^bA^	50.92 ± 0.73 ^aAB^	50.37 ± 0.54 ^aB^	50.21 ± 0.16 ^aB^
	RCC	51.88 ± 0.03 ^bA^	52.45 ± 0.80 ^aA^	51.49 ± 1.03 ^aA^	52.33 ± 1.39 ^aA^
Fat (%)	RC	25.25 ± 0.35 ^aA^	24.25 ± 0.35 ^abA^	24.75 ± 0.35 ^aA^	24.75 ± 0.35 ^aA^
	RCOB	24.50 ± 0.00 ^bA^	24.50 ± 0.71 ^aA^	22.90 ± 0.85 ^abA^	24.25 ± 0.35 ^aA^
	RCC	24.00 ± 0.00 ^bA^	22.75 ± 0.35 ^bAB^	21.50 ± 1.41 ^bB^	23.75 ± 0.35 ^aA^
Fat in dry matter (%)	RC	47.10 ± 0.88 ^aA^	46.77 ± 1.22 ^aA^	47.19 ± 0.93 ^aA^	48.28 ± 0.81 ^aA^
	RCOB	47.13 ± 0.25 ^aA^	48.13 ± 2.08 ^aA^	45.47± 2.17 ^aA^	48.29± 0.56 ^aA^
	RCC	46.26 ± 0.03 ^aA^	43.38 ± 1.33 ^aA^	41.79 ± 3.58 ^aA^	45.41 ± 1.88 ^aA^
Protein (%)	RC	25.35 ± 1.09 ^aA^	21.83 ± 0.23 ^aBC^	20.52 ± 0.41 ^aC^	22.30 ± 0.06 ^bB^
	RCOB	23.59 ± 0.32 ^aAB^	22.50 ± 0.38 ^aB^	24.44 ± 0.74 ^aA^	22.76 ± 0.34 ^abB^
	RCC	25.35 ± 0.28 ^aA^	22.81 ± 1.15 ^aA^	24.83 ± 1.32 ^aA^	23.35 ± 0.30 ^aA^
pH	RC	5.78 ± 0.00 ^aA^	5.26 ± 0.00 ^aD^	5.30 ± 0.00 ^bC^	5.55 ± 0.00 ^aB^
	RCOB	5.52 ± 0.00 ^bA^	5.04 ± 0.00 ^bD^	5.27 ± 0.00 ^cC^	5.37 ± 0.00 ^bB^
	RCC	5.39 ± 0.00 ^cA^	4.89 ± 0.00 ^cD^	5.32 ± 0.00 ^aC^	5.34 ± 0.00 ^cB^
Ash (%)	RC	2.37 ± 0.44 ^aA^	2.94 ± 0.87 ^aA^	3.36 ± 0.84 ^aA^	3.16 ± 0.04 ^aA^
	RCOB	2.85 ± 0.53 ^aA^	2.55 ± 0.06 ^aA^	2.92 ± 0.65 ^aA^	3.09 ± 0.30 ^aA^
	RCC	2.51 ± 0.23 ^aA^	3.26 ± 0.18 ^aA^	3.13 ± 0.16 ^aA^	3.39 ± 1.00 ^aA^
Sodium (g/100 g)	RC	0.43 ± 0.17 ^aA^	0.59 ± 0.23 ^aA^	0.83 ± 0.30 ^aA^	0.67 ± 0.01 ^aA^
	RCOB	0.60 ± 0.12 ^aA^	0.49 ± 0.02 ^aA^	0.72 ± 0.31 ^aA^	0.66 ± 0.07 ^aA^
	RCC	0.45 ± 0.10 ^aA^	0.71 ± 0.08 ^aA^	0.70 ± 0.06 ^aA^	0.73 ± 0.38 ^aA^
Sodium chloride (g/100 g)	RC	1.08 ± 0.41 ^aA^	1.48 ± 0.57 ^aA^	2.06 ± 0.78 ^aA^	1.66 ± 0.02 ^aA^
	RCOB	1.49 ± 0.30 ^aA^	1.21 ± 0.05 ^aA^	1.80 ± 0.78 ^aA^	1.66 ± 0.16 ^aA^
	RCC	1.14 ± 0.25 ^aA^	1.78 ± 0.21 ^aA^	1.76 ± 0.15 ^aA^	1.83 ± 0.95 ^aA^
Water activity	RC	0.954 ± 0.004 ^aAB^	0.945 ± 0.006 ^bB^	0.956 ± 0.001 ^aA^	0.951 ± 0.001 ^bAB^
	RCOB	0.953 ± 0.004 ^aB^	0.956 ± 0.001 ^bB^	0.967 ± 0.004 ^aA^	0.959 ± 0.001 ^aB^
	RCC	0.961 ± 0.001 ^aAB^	0.969 ± 0.002 ^aA^	0.956 ± 0.005 ^aB^	0.954 ± 0.003 ^abB^

The results are presented as means ± SD. Values within the same column marked with different lowercase superscript letters (^a,b,c^) indicate statistically significant differences (*p* < 0.05) between different rolled cheeses. Values within the same row marked with different uppercase letters (^A,B,C,D^) indicate statistically significant differences (*p* < 0.05) among storage time for the same cheese.

**Table 6:** Sensory properties of rolled cheese variants (plain rolled cheese as the control (RC), rolled cheese with a mixture of equal parts of dried oregano and basil (RCOB), and rolled cheese with dried chives (RCC)) at 0, 20, 40, and 60 days of storage.

Parameters	Rolled Cheese	Storage Time (Days)			
		0	20	40	60
Appearance	RC	4.60 ± 0.55 ^A^	4.20 ± 0.45 ^AB^	4.00 ± 1.00 ^AB^	3.20 ± 0.84 ^B^
	RCOB	4.80 ± 0.45 ^A^	4.60 ± 0.55 ^A^	4.20 ± 0.45 ^AB^	3.60 ± 0.55 ^B^
	RCC	4.60 ± 0.55 ^A^	4.20 ± 0.84 ^AB^	4.00 ± 0.71 ^AB^	3.40 ± 0.55 ^B^
Color	RC	5.00 ± 0.00 ^A^	4.80 ± 0.45 ^A^	5.00 ± 0.00 ^A^	4.60 ± 0.55 ^A^
	RCOB	5.00 ± 0.00 ^A^	5.00 ± 0.00 ^A^	4.80 ± 0.45 ^A^	4.60 ± 0.55 ^A^
	RCC	5.00 ± 0.00 ^A^	4.80 ± 0.45 ^A^	4.80 ± 0.45 ^A^	4.40 ± 0.89 ^A^
Texture	RC	5.00 ± 0.00 ^A^	4.80 ± 0.45 ^A^	4.20 ± 0.84 ^A^	3.00 ± 0.71 ^B^
	RCOB	5.00 ± 0.00 ^A^	4.80 ± 0.45 ^A^	4.00 ± 0.71 ^B^	3.20 ± 0.45 ^C^
	RCC	5.00 ± 0.00 ^A^	4.60 ± 0.55 ^AB^	4.00 ± 1.00 ^BC^	3.20 ± 0.84 ^C^
Aroma	RC	5.00 ± 0.00 ^A^	4.80 ± 0.45 ^A^	4.20 ± 0.45 ^B^	3.80 ± 0.45 ^B^
	RCOB	5.00 ± 0.00 ^A^	5.00 ± 0.00 ^A^	4.80 ± 0.45 ^A^	4.20 ± 0.45 ^B^
	RCC	5.00 ± 0.00 ^A^	5.00 ± 0.00 ^A^	4.60 ± 0.89 ^AB^	4.00 ± 0.71 ^B^
Taste	RC	4.80 ± 0.45 ^A^	4.80 ± 0.45 ^A^	4.00 ± 0.71 ^A^	3.00 ± 0.71 ^B^
	RCOB	5.00 ± 0.00 ^A^	5.00 ± 0.00 ^A^	4.80 ± 0.45 ^A^	3.60 ± 0.55 ^B^
	RCC	5.00 ± 0.00 ^A^	5.00 ± 0.00 ^A^	4.60 ± 0.55 ^A^	3.40 ± 0.55 ^B^

The results are presented as means ± SD. Values within the same row marked with different uppercase letters (^A,B,C^) indicate statistically significant differences (*p* < 0.05) among storage time for the same cheese.

## Data Availability

The original contributions presented in the study are included in the article, further inquiries can be directed to the corresponding author.
